# Fast generation of W states of superconducting qubits with multiple Schrödinger dynamics

**DOI:** 10.1038/srep36737

**Published:** 2016-11-09

**Authors:** Yi-Hao Kang, Ye-Hong Chen, Qi-Cheng Wu, Bi-Hua Huang, Jie Song, Yan Xia

**Affiliations:** 1 Department of Physics, Fuzhou University, Fuzhou 350002, China; 2 Department of Physics, Harbin Institute of Technology, Harbin 150001, China

## Abstract

In this paper, we present a protocol to generate a W state of three superconducting qubits (SQs) by using multiple Schrödinger dynamics. The three SQs are respective embedded in three different coplanar waveguide resonators (CPWRs), which are coupled to a superconducting coupler (SCC) qubit at the center of the setups. With the multiple Schrödinger dynamics, we build a shortcuts to adiabaticity (STA), which greatly accelerates the evolution of the system. The Rabi frequencies of the laser pulses being designed can be expressed by the superpositions of Gaussian functions via the curves fitting, so that they can be realized easily in experiments. What is more, numerical simulation result shows that the protocol is robust against control parameters variations and decoherence mechanisms, such as the dissipations from the CPWRs and the energy relaxation. In addition, the influences of the dephasing are also resisted on account of the accelerating for the dynamics. Thus, the performance of the protocol is much better than that with the conventional adiabatic passage techniques when the dephasing is taken into account. We hope the protocol could be implemented easily in experiments with current technology.

Entanglement plays a significant role in quantum information processing (QIP)^1^,^2^,^3^,^4^,^5^,^6^,^7^,^8^,^9^,^10^,^11^,^12^. Therefore, the generation of entangled states for two or more particles is not only fundamental for showing quantum nonlocality^13^,^14^,^15^, but also useful in many research fields in QIP, such as, quantum secure direct communication^16^,^17^, quantum secret sharing^18^,^19^, quantum teleportation^20^,^21^, quantum cloning machine^22^,^23^ and so on. For multi-qubit entanglement, there are two major types of entangled states, the W states^14^ and the Greenberger-Horne-Zeilinger (GHZ) states^15^, which can not be converted to each other by local operations and classical communications. The GHZ states are usually called as “maximally entangle” in several senses, e.g., the GHZ state violates Bell inequalities maximally. But a particle trace of a GHZ state results in a maximally mixed state compared with a nonmaximally mixed result for a W state, i.e., the W states show perfect correlations. Therefore, in past several years, the W states have attracted more attentions because of their robustness against qubit loss and advantages in quantum teleportation^20^.

Till now, the generation of the W states has been studied in numerous systems^24^,^25^,^26^,^27^,^28^,^29^,^30^,^31^,^32^,^33^,^34^,^35^, such as the atom-cavity coupled systems^24^,^25^,^26^, electronic spin qubits inside the quantum dots systems^27^, photons and linear optical systems^28^,^29^, superconducting qubits (SQs) systems^30^,^31^,^32^,^33^,^34^,^35^,^36^, etc. Among of these protocols^24^,^25^,^26^,^27^,^28^,^29^,^30^,^31^,^32^,^33^,^34^,^35^,^36^, the generation of W states with SQs has shown fantastic advantages, since new progress in circuit cavity quantum electrodynamics makes it a standout performance among the most promising candidates for implementing QIP^37^,^38^. It has been shown that, the SQs (e.g., flux, phase and charge qubits) and microwave resonators can be manufactured using modern integrated circuit technology, their features can be characterized and adjusted *in situ*. Moreover, the SQs have relatively long decoherence times^39^, and various single and multiple qubits operations with state readout have been shown^40^,^41^,^42^,^43^. Furthermore, a superconducting resonator can provide a quantized cavity field, in order that the fast and long-range interaction between distant SQs could be mediated^44^,^45^,^46^. What is more, it has been proved by both a lot of theoretical researches^47^,^48^ and experiments^49^,^50^ that, the strong-coupling limit can be easily realized with SQs. Therefore, creating W states with SQs is a wise choice.

On the other hand, if one decides to generate W states with SQs, another question is how to accurately controlling the system with high fidelity. Many previous researches have indicated that the adiabatic passages, especially the stimulated Raman scattering involving adiabatic passage (STIRAP) and its variants^51^,^52^,^53^,^54^ hold robustness against variations of the controlled parameters. Generally speaking, if the system remains in the instantaneous ground state of its time-dependent Hamiltonian during the whole evolution process under an adiabatic control, the dissipations caused by decoherence, noise and losses may be repressed. However, we all know that, to prevent the transition between each instantaneous eigenstate, the adiabatic condition is required, which will badly limit the evolution speed of the system. During a long evolution, the dissipations may accumulate and finally destroy the intended dynamics. For example, refs 31 and 36 has shown that, the fidelity for generating of the W state by using adiabatic passage is quite sensitive to the dephasing, which is an ineluctable element of the decoherence mechanisms in the superconducting systems, i.e., a small increase of the dephasing rates causes a large decrease of the fidelity; this will also bring challenges to the experiments. Therefore, to overcome the problem causing by the long evolution time of the adiabatic passage, one should speed up the evolution by using some other techniques. To speed up the evolution, using resonant interaction is a choice. But unfortunately, using resonant interaction will make the system quite sensitive to the variations of experimental parameters. For example, if there are a little variations of the evolution time or Rabi frequencies of laser pulses, the fidelity will decrease a lot. It is also proved in ref. 32 that, with resonant interaction, the population of each state changes rapidly when the evolution time increases, and a high fidelity of the target state only appears in very narrow ranges around some certain moments. Therefore, methods with both robustness and high speed are desired, and consequently, a new technique called “Shortcuts to adiabatic passage” (STAP) has been proposed^55^,^56^,^57^,^58^,^59^,^60^,^61^,^62^,^63^.

The STAP is closely related to adiabatic passage but totally breaks the limit of the adiabatic condition. It depicts a rapid adiabatic-like process which is not really adiabatic but leading to the same goals with adiabatic process. With these advantages, the STAP has attracted a lot of interests and been used in many research fields including “fast cold-atom”, “fast ion transport”, “fast quantum information processing”, “fast wave-packet splitting”, “fast expansion”, and so on refs 64, 65, 66, 67, 68, 69, 70, 71, 72, 73, 74, 75, 76, 77, 78, 79, 80, 81, 82, 83, 84, 85, 86, 87, 88, 89, 90, 91, 92, 93. Among of these protocols^55^,^56^,^57^,^58^,^59^,^60^,^61^,^62^,^63^,^64^,^65^,^66^,^67^,^68^,^69^,^70^,^71^,^72^,^73^,^74^,^75^,^76^,^77^,^78^,^79^,^80^,^81^,^82^,^83^,^84^,^85^,^86^,^87^,^88^,^89^,^90^,^91^,^92^,^93^, the method named “Transitionless quantum driving” (TQD)^58^,^59^,^60^,^61^ has shown its power to construct the STAP. However, when we accelerate adiabatic protocols using TQD, the structure or the values of the shortcut-driving Hamiltonian might not exist in practice. For example, in refs 24,80,94, 95, 96, the authors did a lot to design Hamiltonians to overcome the problem caused by the problematic terms which are actually equivalent to the special one-photon 1–3 pulse (the microwave field). Nevertheless, the operations usually cause some other problems or make some other limiting conditions to the protocols, for examples, there will be a limiting condition for the total operation time to generate the entangled states. Therefore, numerous protocols with different methods^97^,^98^,^99^,^100^,^101^,^102^,^103^,^104^,^105^,^106^,^107^ have been further presented to avoid the problematic terms of the system’s Hamiltonian which is designed by TQD. Among of these methods^97^,^98^,^99^,^100^,^101^,^102^,^103^,^104^,^105^,^106^,^107^, the multiple Schrödinger dynamics^104^,^105^ is a very interesting method. It exploits iterative interaction pictures to obtain Hamiltonians with physically feasible structure for quantum systems. Moreover, by choosing suitable boundary conditions, it enables the designed interaction picture to reproduce the same final population (or state) as those in the original Schrödinger picture. In 2012, Ibáñez *et al.*^105^ have adopted some Schrödinger pictures and dynamics to design alternative and feasible STAP for harmonic transport, trap expansions and trap compressions. Subsequently, in 2013, Ibáñez *et al.*^104^ have studied the capabilities and limitations of superadiabatic iterations to construct a sequence of shortcuts to adiabaticity by iterative interaction pictures. Afterwards, Song *et al.*^106^ have investigated the physical feasibility of the multiple Schrödinger dynamics in a three-level systems, and obtained very interesting results in 2016. They have shown that the Hamiltonian of the interaction picture in the second iteration has the same form as the Hamiltonian in the original Schrödinger picture. This makes the multiple Schrödinger dynamics useful in three-level systems.

Inspired by the protocols in refs 104, 105, 106, as well as considering the advantages of the superconducting systems, we come up with a protocol for generating a W state of three SQs by using multiple Schrödinger dynamics. With the help of the multiple Schrödinger dynamics, a STAP is constructed, which greatly speeds up the evolution of the system. The Hamiltonian being designed in this protocol has the same form as the system’s original Hamiltonian. Moreover, the Rabi frequencies of the laser pulses being designed can be expressed by the superpositions of Gaussian functions assisted by the curves fitting, so that they can be realized in experiments. Numerical simulation demonstrates that the protocol is robust against control parameters variations and decoherence mechanisms, such as the dissipations from the coplanar waveguide resonators (CPWRs) and the energy relaxation of SQs. What is more, the influences of the dephasing are also resisted because of the accelerating for the dynamics. Therefore, the performance of the protocol is much better comparing with the conventional adiabatic passage techniques when the dephasing is taken into account. Based on a circuit quantum electrodynamics system, the protocol could be controlled and implemented readily in experiments.

The article is organized as follows. In the section of “The multiple Schrödinger dynamics”, we will introduce the method of the multiple Schrödinger dynamics. In the section of “Fast generation of W states of superconducting qubits with multiple Schrödinger dynamics”, we will describe the generation of a W state of three SQs in detail. In the section of “Numerical simulations and discussions”, we will investigate the performance of the protocol when the control parameters variations and decoherence mechanisms are considered. Finally, the conclusions will be given in the section of “Conclusions”.

## The multiple Schrödinger dynamics

In this section, we would like to review the multiple Schrödinger dynamics^104^,^105^,^106^ firstly. Assume that the original Hamiltonian of the system is *H*_0_(*t*). We perform a picture transformation as 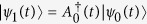, where 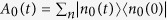 and |*n*_0_(*t*)〉 is the *n*-th instantaneous eigenstate of *H*_0_(*t*). So, the Hamiltonian in the 1-st interaction picture is 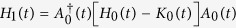 with 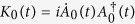. Suppose that the 1-st modified Schrödinger Hamiltonian is 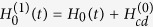. If one hopes the transitions between instantaneous eigenstates {|*n*_0_(*t*)〉} are all forbidden, the simplest choice is 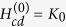, so that the Hamiltonian in the 1-st interaction picture is diagonal. If the 1-st modified Hamiltonian 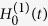 is difficult to be realized, the 2-nd interaction picture should be introduced. Assume that {|*n*_1_(*t*)〉} are the eigenstates of *H*_1_(*t*). We perform a picture transformation as 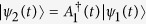 with 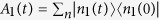. Then, we obtain the Hamiltonian in the 2-nd interaction picture as 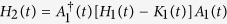 with 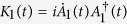. Suppose that the 2-nd modified Schrödinger Hamiltonian is 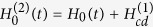. To forbid the transitions between {|*n*_1_(*t*)〉} and diagonalize the Hamiltonian in the 2-nd interaction picture, 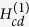 can be calculated as 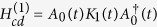. Repeating the processing as the 1-st and the 2-nd iterations, according to the Hamiltonian in the *j*-th (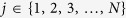) interaction picture (*H*_*j*_(*t*)), one can obtain the *j*-th modified Schrödinger Hamiltonian as
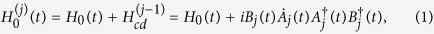
where 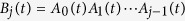 and 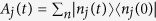 with {|*n*_*j*_(*t*)〉} being the instantaneous eigenstates of *H*_*j*_(*t*). Governed by the Hamiltonian 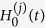, the transitions between instantaneous eigenstates {|*n*_*j*_(*t*)〉} of *H*_*j*_ are forbidden.

## Fast generation of W states of superconducting qubits with multiple Schrödinger dynamics

**Figure 1:**
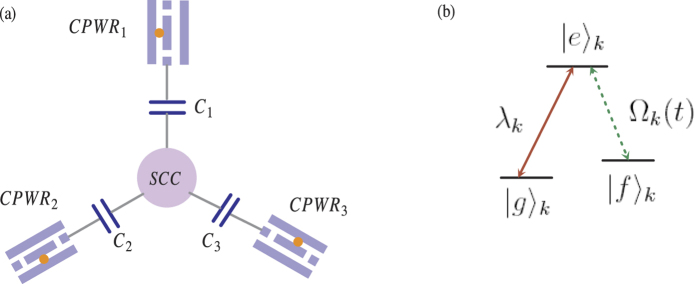
(**a**) Schematic diagram of three CPWRs and a SCC qubit (a circle at the center). (**b**) The energy-level structure of *SQ*_*k*_.

In this section, we will show how to generate a W state of three SQs with multiple Schrödinger dynamics. Consider a system composed of a superconducting coupler (SCC) qubit and three CPWRs (*CPWR*_1_, *CPWR*_2_ and *CPWR*_3_). As shown in Fig. 1(a), the SCC qubit in the center of the devices is coupled to *CPWR*_*k*_ through capacitor *C*_*k*_ (*k* ∈ {1, 2, 3}). There is a SQ named *SQ*_*k*_ in the *CPWR*_*k*_, which has an excited state |*e*〉_*k*_ and two ground states |*g*〉_*k*_ and |*f*〉_*k*_. As shown in Fig. 1(b) the transition |*e*〉_*k*_ ↔ |*f*〉_*k*_ is driven by the laser pulse with Rabi frequency Ω_*k*_(*t*), and the transition |*e*〉_*k*_ ↔ |*g*〉_*k*_ is coupled to *CPWR*_*k*_ with coupling constant *λ*_*k*_. As for the SCC qubit, it has an excited state |*e*〉_*c*_ and two ground states |*g*〉_*c*_ and |*f*〉_*c*_, which has similar structure as the three SQs. The transition |*e*〉_*c*_ ↔ |*f*〉_*c*_ is driven by the laser pulse with Rabi frequency Ω_*c*_(*t*). Different from the three SQs, the transition |*e*〉_*c*_ ↔ |*g*〉_*c*_ may couple to three CPWRs with different coupling constants. We assume that the coupling constant for the transition |*e*〉_*c*_ ↔ |*g*〉_*c*_ coupled to *CPWR*_*k*_ is *ν*_*k*_. Therefore, in the interaction picture, the Hamiltonian for the system can be written by
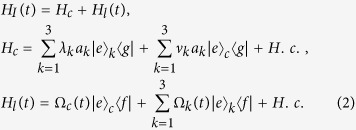


For simplicity of calculations, we adopt *λ*_*k*_ = *λ* and *ν*_*k*_ = *ν* in the following. Assuming that the initial state of the system is 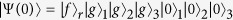, where, |0〉_*k*_ and |1〉_*k*_ are the vacuum state and one-photon state of the cavity mode in *k*-th CPWR, respectively. The excited number operator of the system is defined by 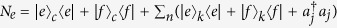. As [*N*_*e*_, *H*_*I*_] = 0, and 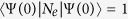, the system will remain in the one-excited subspace spanned by
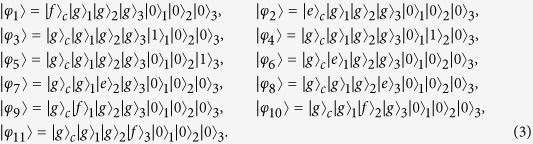


Moreover, the eigenstates of *H*_*c*_ can be described as
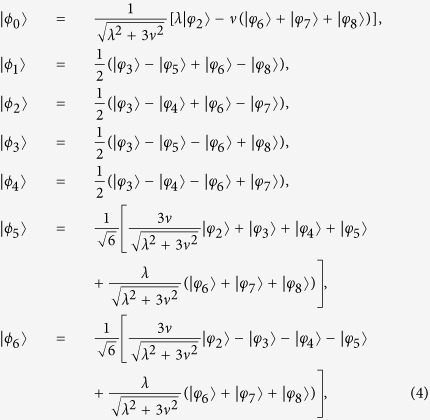
with corresponding eigenvalues *ε*_0_ = 0, *ε*_1_ = *λ*, *ε*_2_ = *λ*, *ε*_3_ = −*λ*, *ε*_4_ = −*λ*, 
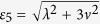
 and 
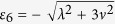
, respectively.

For simplicity, we set 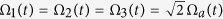 and 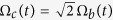. Under the condition Ω_*a*_(*t*), 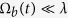, *ν*, we can obtain the effective Hamiltonian of the system as
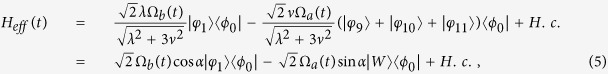
where 
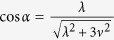
, 
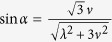
, 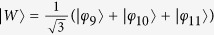. Without loss the generality, we assume *α* = *π*/4. We also assume 
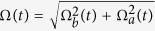
 and 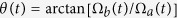. Then, the system’s effective Hamiltonian can written by
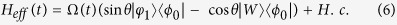


Afterwards, the instantaneous eigenstates of *H*_*eff*_(*t*) can be solved as
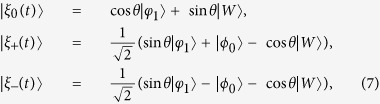
with corresponding eigenvalues *ϵ*_0_ = 0, *ϵ*_+_ = Ω and *ϵ*_−_ = −Ω, respectively. Therefore, the picture transformation for the 1-st iteration in basis 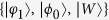 is
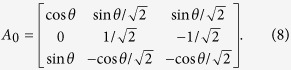


By calculating 
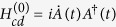
, in basis 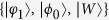, we obtain
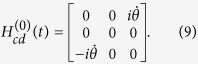


If we add 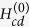 to modify the Hamiltonian *H*_*eff*_(*t*) in Eq. (6), the structure of the system is also required to be adjusted. Therefore, we consider the 2-nd iteration picture to find another shortcut. Then, the Hamiltonian in the 1-st iteration picture can be solved in basis 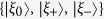 as
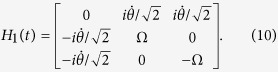


Defining 
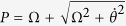
, 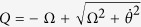 and 
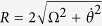
, the eigenstates of *H*_1_(*t*) can be described as
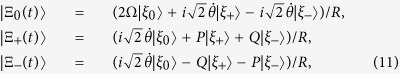
corresponding to the eigenvalues *η*_0_ = 0, 
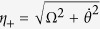
 and 
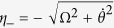
, respectively. Therefore, the picture transformation for the 2-nd iteration in basis 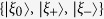 can be given by
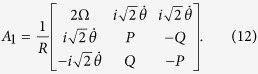


Submitting *j* = 2, Eqs (6) and (12) into Eq. (1), one can obtain the 2-nd modified Hamiltonian for *H*_*eff*_(*t*) in basis 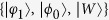 as
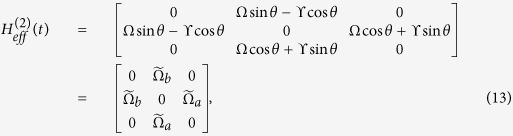
where 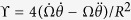, 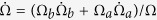, 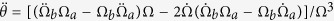, 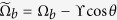 and 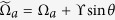. We find that 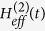 has the same form as *H*_*eff*_(*t*). Therefore, using 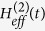 instead of *H*_*eff*_(*t*), we only need to adjust the Rabi frequencies Ω_*b*_(*t*) and Ω_*a*_(*t*).

Now, let us design the frequencies Ω_*b*_(*t*) and Ω_*a*_(*t*) so that the system governed by 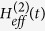 can be driven from its initial state 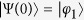 to the target state |*W*〉. Firstly, when the system is governed by 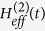, the transitions between instantaneous eigenstates 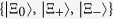 of *H*_1_(*t*) are forbidden. Assuming that the initial time is *t*_*i*_ = 0 and the final time is *t*_*f*_ = *T*, we find that if the boundary condition 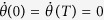 is satisfy, the instantaneous eigenstate 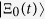 of *H*_1_ will coincide with the dark state 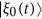 of *H*_*eff*_(*t*) at *t* = 0 and *t* = *T*. Therefore, we adopt the boundary condition 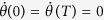, and we set *θ*(0) = 0 and *θ*(*T*) = *π*/2. Then, we will have the following results
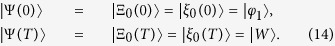


**Figure 2:**
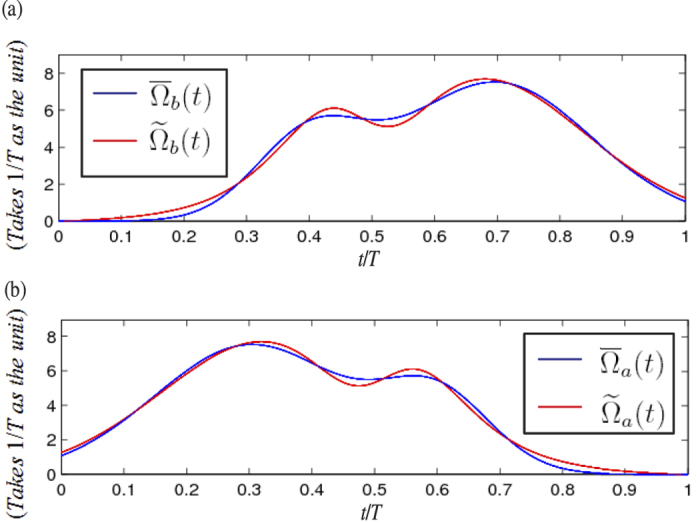
(**a**) Comparison between 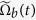 and 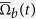 (versus *t*/*T*). (**b**) Comparison between 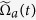 and 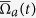 (versus *t*/*T*).

So, the system will evolve along the instantaneous eigenstate 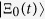 of *H*_1_ and finally at *t* = *T*, we can obtain the target state 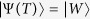. After the boundary conditions of *θ* and 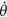 are set, in the second step, let us design the Rabi frequencies of the laser pulses. To satisfy the boundary conditions of *θ* and 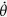 mentioned above, we firstly design the Ω_*b*_ and Ω_*a*_ via STIRAP. Ω_*b*_ and Ω_*a*_ can be expressed as
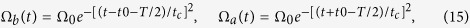
where Ω_0_ is the pulse amplitude, *t*_0_ = 0.16*T* and *t*_*c*_ = 0.25*T* are two related parameters. By calculating 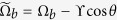 and 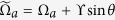, one can obtain the Rabi frequencies 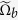 and 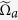 of laser pulses for the modified Hamiltonian 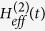. However, the forms of 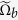 and 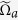 are too complex to be realized in experiments. For the sake of making the protocol more feasible in experiments, the Rabi frequencies of laser pulses should be expressed by some frequently used functions (e.g. Gaussian functions and sine function), or their superpositions. Fortunately, by using curves fitting, 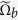 and 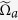 can be replaced respectively with 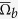 and 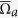, whose expressions can be written by
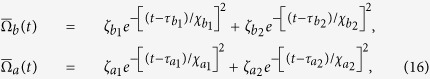


where,


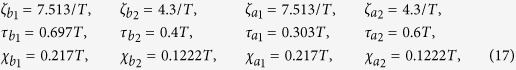
when Ω_0_ = 8/*T*. As a comparison, we plot 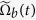 and 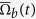 versus *t*/*T* in Fig. 2(a) and 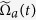 and 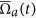 versus *t*/*T* in Fig. 2(b). As shown in Fig. 2, the curve for 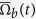 (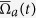) is very close to that for 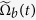 (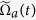). In the next section, we will show that the laser pulses with Rabi frequencies 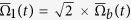, 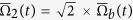, 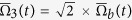 and 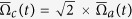 can drive the system from its initial state 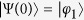 to the target state 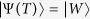 with a high fidelity, so, the replacements here for the Rabi frequencies of the laser pulses are effective.

## Numerical Simulations and Discussions

**Figure 3:**
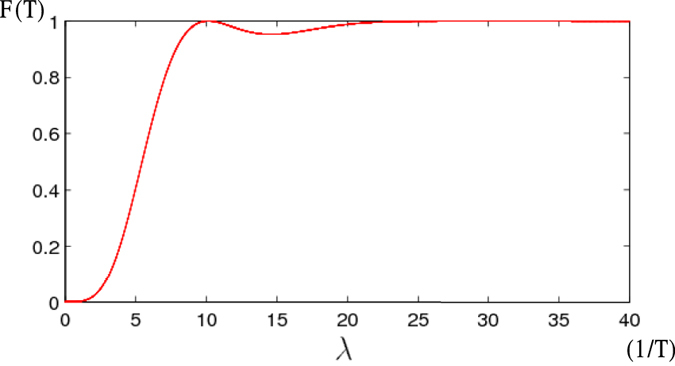
The final fidelity *F*(*T*) versus *λ*.

In this section, various numerical simulations will be performed to demonstrate the effective of the present protocol. The fidelity of the target state |*W*〉 is defined as 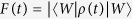, where *ρ*(*t*) is the density operator of the system. Firstly, let us choose suitable coupling constants *λ* and *ν*. As we adopted *α* = *π*/4, the relation between *λ* and *ν* is 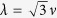. And the Rabi frequencies of laser pulses satisfy 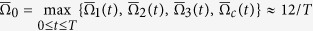. We plot the final fidelity *F*(*T*) versus *λ* in Fig. 3. As shown in Fig. 3, *F*(*T*) is near 1 around *λ* = 10/*T*. Moreover, *F*(*t*) is close to 1 when *λ* > 20/*T*. One can easily find that even when the condition 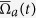, 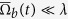, *ν* is not satisfied well, the target state |*W*〉 can also be obtained. This can also easily be understood, as the evolution of the system, between the initial state 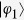 and the target state |*W*〉, may move along different medium states, and it is not governed by the effective Hamiltonian *H*_*eff*_(*t*), which guides the system moving through the dark state 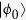 of *H*_*c*_ as the only medium state. However, when the condition 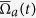, 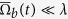, *ν* is broken, the system may move through a medium state with higher energy. That will make the evolution of the system suffers more from dissipations. On the other hand, for a relative higher evolution speed, the value of *λT* should not be too large, as *λ* has a upper limit in a real experiment. Therefore, to make the protocol with both high speed and robustness against dissipations, we choose *λ* = 35/*T*, slightly larger than 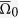 (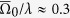).

**Figure 4:**
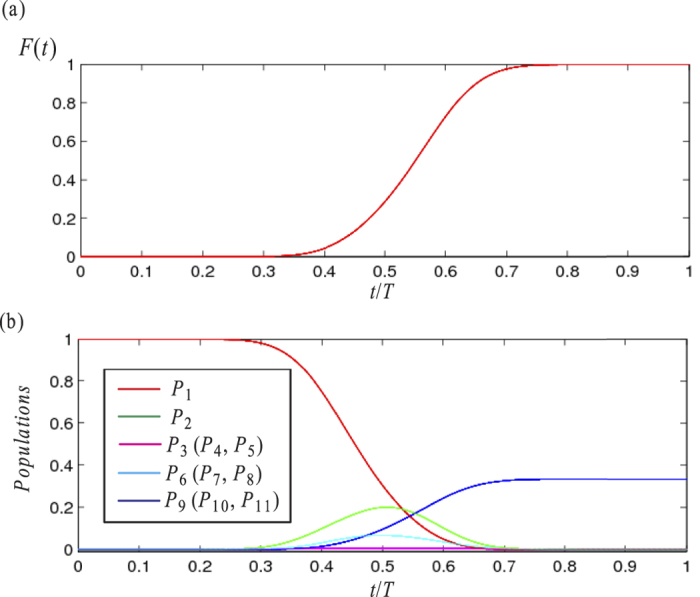
(**a**) The fidelity *F*(*t*) versus *t*/*T*. (**b**) The population *P*_*m*_ of 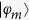 versus *t*/*T*.

Secondly, since we have adopted a suitable value of the coupling constant *λ*, we would like to examine the fidelity *F*(*t*) and the population 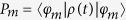 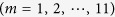 of state 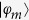 during the evolution. The fidelity *F*(*t*) versus *t*/*T* is plotted in Fig. 4(a). And the the population *P*_*m*_ of each state is shown in Fig. 4(b). As shown in Fig. 4(a), the fidelity *F*(*t*) keeps steady during time intervals [0, 0.3*T*] and [0.8*T*, *T*], and increases rapidly to approach 1 during time interval [0.3*T*, 0.8*T*]. As shown in Fig. 4(b), *P*_1_ falls from 1 to 0 during evolution; *P*_9_, *P*_10_ and *P*_11_ are initial 0 and final 1/3 at time *t* = *T* as our expectation.

**Figure 5:**
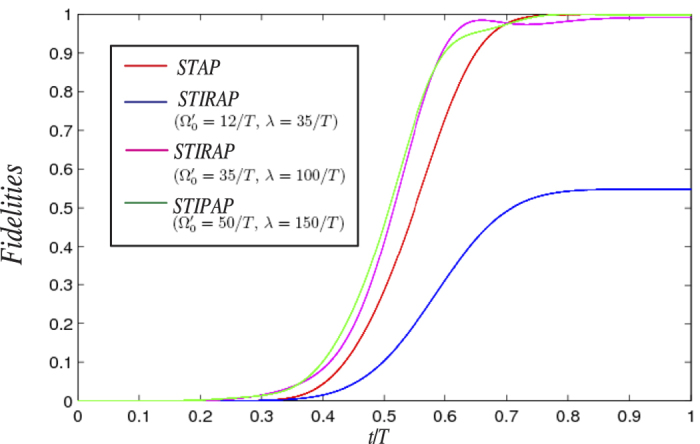
The fidelities of the target state |*W*〉 versus *t*/*T* with different methods.

Thirdly, to show that the present protocol is faster than the adiabatic protocol, we plot the fidelity of the target state |*W*〉 with different methods versus *t*/*T* in Fig. 5. The Rabi frequencies of laser pulses for the STIPAP method can be set as 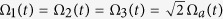 and 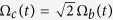, where Ω_*a*_(*t*) and Ω_*b*_(*t*) are shown in Eq. (15). And it is easy to obtain that 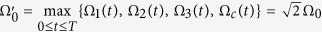. As shown in Fig. 5, the curve of “STAP” describes the change of the fidelity versus *t*/*T* of the present protocol, and the curves of “STIRAP” describe the changes of fidelities versus *t*/*T* of the STIRAP method under some different conditions. Seen from blue line of Fig. 5, if one use STIRAP method with the same condition as the present protocol (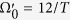, *λ* = 35/*T*), the final fidelity is only about 0.55 due to the greatly violation of the adiabatic condition. Even when 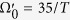, *λ* = 100/*T* (see the pink line of Fig. 5) for the STIRAP method, the fidelity can get close to 1, but the result is still a little unsatisfactory. When 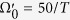, *λ* = 150/*T* (see the green line of Fig. 5) for the STIRAP method, the fidelity can approach 1. However, in this case, the laser amplitude 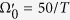 is much larger than the one (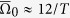) of the present protocol. If one desire a relative high evolution speed, the product (denotes by *μ*) of laser amplitude and the evolution time is the smaller the better. Because when two persons have the same value of the laser amplitudes, the one with smaller *μ* will have less evolution time. Therefore, comparing with the STIRAP method, the present protocol to obtain a W state is much faster by using multiple Schrödinger dynamics. In addition, it is also been shown in ref. 31 that, to obtain a W state with the adiabatic passage with a fidelity larger that 0.99, the authors should chose *λ* > 100/*T* and Ω_0_ = 0.35*λ*. That supports the discussion here as well.

**Figure 6:**
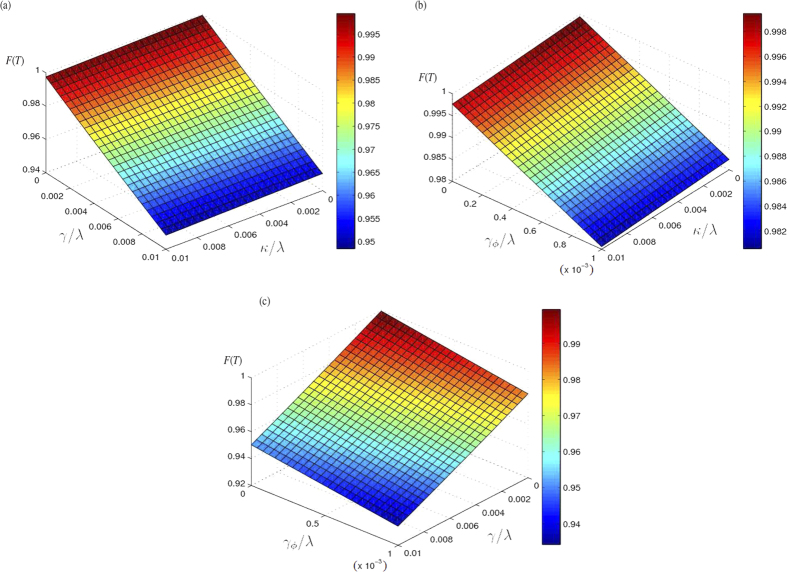
(**a**) The final fidelity *F*(*T*) versus 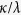 and *γ*/*λ*. (**b**) The final fidelity *F*(*T*) versus 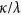 and 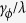. (**c**) The final fidelity *F*(*T*) versus *γ*/*λ* and 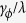.

Fourthly, since the dissipations caused by decoherence mechanisms are ineluctable in real experiments, it is worthwhile to discuss the fidelity *F*(*t*) when different kinds of decoherence factors are considered. In the present protocol, the decay of the cavity mode in each CPWR, the energy relaxation and the dephasing of every SQ play the major roles. The evolution of the system can be described by a master equation in Lindblad form as following
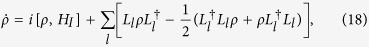
where, *L*_*l*_ (*l* = 1, 2, 3, …, 19) is the Lindblad operator. There are nineteen Lindblad operators
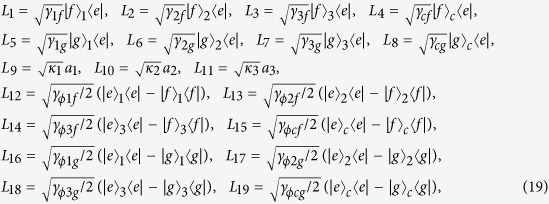
in which *γ*_*ks*_ and 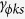 (*k* = 1, 2, 3, *s* = *f*, *g*) are the energy relaxation rate and dephasing rate of the *k*-th SQ for decay path |*e*〉_*k*_ → |*s*〉_*k*_, respectively. And *γ*_*cs*_ and 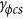 (*s* = *f*, *g*) are the energy relaxation rate and dephasing rate of the SCC qubit for decay path |*e*〉_*c*_ → |*s*〉_*c*_, respectively. 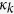 (*k* = 1, 2, 3) is the decay rate of the *k*-th cavity mode in *CPWR*_*k*_. We suppose *γ*_*ks*_ = *γ*, 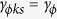 (*k* = 1, 2, 3, *s* = *f*, *g*) and 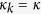 (*k* = 1, 2, 3) for simplicity. We plot the final fidelity *F*(*T*) versus 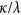 and *γ*/*λ* in Fig. 6(a), versus 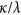 and 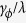 in Fig. 6(b) and versus *γ*/*λ* and *γ*/*λ* in Fig. 6(c). And we also examine some samples of the final fidelities *F*(*T*) with corresponding 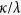, *γ*/*λ* and 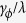 and give them in Table 1. As shown in Fig. 6 and Table 1, we can obtain following results. (i) *F*(*T*) is insensitive to decays of the cavity modes in CPWRs. This is easy to be understood by seeing Fig. 4(b). Because the populations of 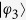, 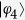 and 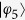 are all almost zero during the whole evolution, the influences from decays of the cavity modes in CPWRs will be greatly resisted. (ii) The fidelity suffers more influence from the energy relaxations of SQs comparing with the influences from decays of the cavity modes in CPWRs. However, *F*(*T*) is 0.9502 when *γ*/*λ* = 0.01, 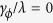 and 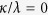, i.e., the decreasing of *F*(*T*) caused by the increasing of *γ* is only about 0.05. Therefore, the present protocol is also robust against the energy relaxations of SQs. (iii) The dephasing plays a significant role here. When 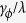 increases from 0 to 0.001, *F*(*T*) decreases from 1 to 0.9824. However, in ref. 31, with the adiabatic passages, the fidelity of the target W state deceases from 1 to 0.85 when 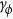 increases only from 0 to 0.0001. This shows that the present protocol is more robust against the dephasing comparing with the adiabatic passages. According to ref. 107, in experiments, parameters *λ* = 2*π* × 300 MHz, *γ* = 6*π* MHZ, 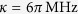 and 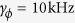 can be realized. By submitting these parameters, we have *F*(*T*) = 0.9484.

**Figure 7:**
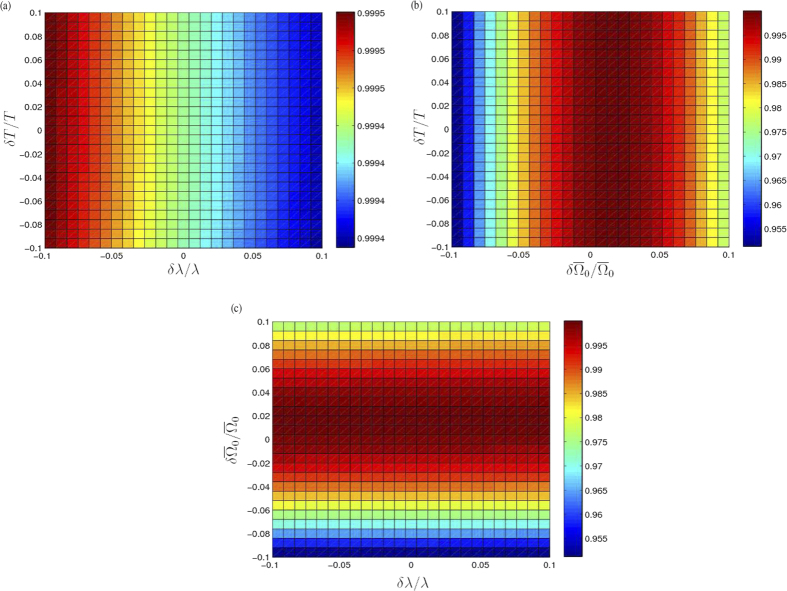
(**a**) The final fidelity *F*(*T*′) versus *δT*/*T* and *δλ*/*λ*. (**b**) The final fidelity *F*(*T*′) versus *δT*/*T* and 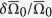. (**c**) The final fidelity *F*(*T*) versus 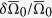 and *δλ*/*λ*.

Fifthly, since most of the parameters are hard to faultlessly achieve in experiments, it is necessary to investigate the variations of the parameters caused by the experimental imperfection. Here, we discuss the variation *δT* of the evolution time *T*, the variation 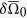 of the laser amplitude 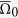 and the variation *δλ* of the coupling constant *λ*. We plot *F*(*T*′) versus *δT*/*T* and *δλ*/*λ* in Fig. 7(a), *F*(*T*′) versus *δT*/*T* and 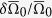 in Fig. 7(b) and *F*(*T*) versus *δλ*/*λ* and 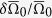 in Fig. 7(c), where *T*′ = *T* + *δT* is the real evolution time when the variation of the evolution time is taken into account. Seen from Fig. 7(a,c), the final fidelity is quite insensitive to the variation *δλ*. This results has also been announced in Fig. 3. Moreover, according to Fig. 7(a,b), the final fidelity *F*(*T*′) is very robust against the variation *δT*. The final fidelity almost unchanged when both *δT*/*T*, *δλ*/*λ* ≤ 10%. As shown in Fig. 7(b,c), variation 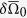 influences the final fidelity mainly. However, even when 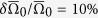, the final fidelity is still higher than 0.95. Therefore, we conclude that the present protocol for generating a W state of three SQs is robust against the variations *δT*, 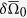 and *δλ*.

**Figure 8:**
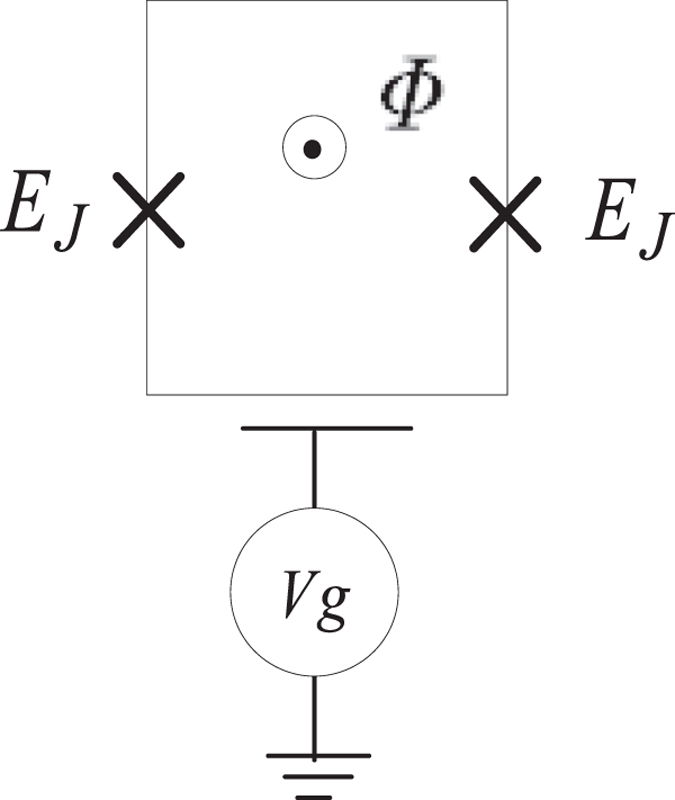
Schematic diagram of a charge qubit.

Sixthly, in experiments, the protocol can be realized in charge qubits and CPWR coupling system. In other words, all the superconducting qubits including the SCC qubit can be chosen to be charge qubits. The structure of the a charge qubit is shown in Fig. 8. As shown in Fig. 8, the charge qubit contains a gate capacitance and two Josephson junctions with Josephson energy *E*_*J*_. The charge qubit can be manipulated by controlling the gate voltage *V*_*g*_ and the magnetic flux 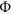 threading the loop. It was pointed out in previous protocols^108^,^109^ that, for a charge qubit with energy structure as Fig. 1(b), when an external applied magnetic flux 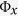 of a pulse threads the ring, it can driven the transition between |*e*〉 ↔ |*f*〉, and the Rabi frequency can be given by


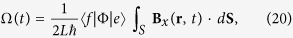


where, *L* is the loop inductance, *S* is surface bounded by the loop of the charge qubit, **B**_*x*_ (**r**, *t*) is the magnetic components of the pulse in the superconducting loop of the charge qubit. For the SQs inside the CPWR, the cavity mode with frequency 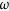 can couples resonantly to the levels |*g*〉 and |*e*〉 and gives the coupling constant as
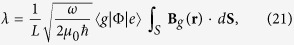
where, **B**_*g*_ (**r**) is the magnetic components of the cavity mode^109^,^110^. For the SCC qubits placed in the center of the devices, it can couple capacitively to three different CPWR directly. This kind of directly coupling has been shown in many previous protocols both in theory^38^,^111^. For example, Yang *et al.*^38^ have used these kind of coupling to generate entanglement between microwave photons and qubits in multiple cavities coupled by a superconducting qubit. Moreover, to improve the efficiency of the coupling between SCC qubit and each CPWR, one can chose SCC qubit to be a transmon^112^ or a phase qubit^113^ as well.

## Conclusions

In conclusion, we have proposed a protocol to generate a W state of three SQs by using multiple Schrödinger dynamics to construct a shortcut to adiabaticity, so that the evolution of the system has been greatly accelerated. Interestingly, the form of the Hamiltonian being designed by the multiple Schrödinger dynamics was the same as that of the system’s original Hamiltonian. Therefore, we only need to adjust the Rabi frequencies of laser pulses. In this protocol, the Rabi frequencies of the laser pulses can be expressed by the superpositions of Gaussian functions via the curves fitting. So, the laser pulses can be realized easily in experiments. One the other hand, numerical simulations results have demonstrated that the protocol is robust against different kinds of control parameters variations and decoherence mechanisms. Notably, the present protocol is more robust against the dephasing, comparing with adiabatic passages. Therefore, we hope the protocol could be controlled and implemented easily in experiments based on a circuit quantum electrodynamics system.

## Additional Information

**How to cite this article**: Kang, Y.-H. *et al.* Fast generation of W states of superconducting qubits with multiple Schrödinger dynamics. *Sci. Rep.* **6**, 36737; doi: 10.1038/srep36737 (2016).

**Publisher’s note**: Springer Nature remains neutral with regard to jurisdictional claims in published maps and institutional affiliations.

## Figures and Tables

**Table 1:** Samples of the final fidelities *F*(*T*) with corresponding 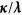 , *γ*/*λ* and 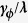 .

*κ*/*λ* (×10^−2^)	*γ*_*ϕ*_/*λ* (×10^−3^)	*γ*/*λ* (×10^−2^)	*F*
1	1	1	0.9325
1	1	0.8	0.9418
1	0.8	1	0.9356
0.8	1	1	0.9328
0.8	0.8	0.8	0.9453
0.8	0.8	0.5	0.9596
0.8	0.5	0.8	0.9502
0.5	0.8	0.8	0.9458
0.5	0.5	0.5	0.9651
0.5	0.5	0.3	0.9749
0.5	0.3	0.5	0.9684
0.3	0.5	0.5	0.9654
0.3	0.3	0.3	0.9786
0.3	0.3	0.1	0.9887
0.3	0.1	0.3	0.9820
0.1	0.3	0.3	0.9790
0.1	0.1	0.1	0.9924
